# Mutismus in Han Kangs „Griechischstunden“ – eine Lektüre aus psychiatrischer Sicht

**DOI:** 10.1007/s00115-025-01817-7

**Published:** 2025-02-26

**Authors:** Katharina Domschke

**Affiliations:** Klinik für Psychiatrie und Psychotherapie, Universitätsklinikum Freiburg, Medizinische Fakultät, Albert-Ludwigs-Universität Freiburg, Hauptstraße 5, 79104 Freiburg, Deutschland

Der Nobelpreis für Literatur 2024 wurde der südkoreanischen Schriftstellerin Han Kang (*1970) verliehen für – wie es in der Begründung der Jury hieß – „ihre intensive Prosa, die sich historischen Traumata stellt und die Zerbrechlichkeit des menschlichen Lebens aufzeigt“ [6]. Einer ihrer Romane, der sich mit einer psychopathologischen Reaktion auf ein persönliches Trauma befasst, trägt den Titel „Griechischstunden“ [5]. Eingebettet in eine Liebesgeschichte wird hier die völlige Aufhebung der Fähigkeit zur mündlichen Sprachproduktion im Sinne eines Mutismus dargestellt. Die namenlos bleibende Protagonistin aus Seoul hat innerhalb weniger Monate sowohl ihre Mutter als auch den Kampf um das Sorgerecht für ihren neunjährigen Sohn und in diesem Zuge auch ihre Stimme verloren. Die Frau nimmt Unterricht in Altgriechisch und entwickelt über die Zeit eine Liebesbeziehung mit ihrem Griechischlehrer, der seinerseits zunehmend erblindet.

Während einer Griechischstunde wird die Symptomatik deutlich: „Bitte versuchen Sie das vorzulesen“ […] „Sie bewegt die Lippen. Mit der Zungenspitze befeuchtet sie die Unterlippe. […] Sie öffnet den Mund und schließt ihn gleich wieder. […] Sie hört und versteht jedes geschriebene Wort ganz genau, aber ihr kommt kein Ton über die Lippen. […] Ihre Mundhöhle ist leer wie der Schacht eines ruhenden Aufzugs oder wie ein Blutgefäß ohne Blut.“ In einer Rückblende in das Frühjahr des Vorjahres wird die Genese der aktuellen Sprachlosigkeit dargestellt: „Ihrer Meinung nach war sie grundlos und aus heiterem Himmel davon überfallen worden. Zugegeben, ihre Mutter war vor sechs Monaten gestorben, und im Zuge ihrer Scheidung vor einigen Jahren hatte sie schließlich das Sorgerecht für ihren neunjährigen Sohn verloren. […] Seitdem litt sie unter Schlaflosigkeit.“ Etwas später im Verlauf des Romans erfährt man von der emotionalen Verfassung der Hauptfigur nach einer Auseinandersetzung mit ihrem Expartner: „So verstummte sie, bis in alle Ewigkeit. Der Hass, der über eine lange Zeit in ihr gebrodelt hatte, schien im Moment ausbalanciert zu sein, ebenso wie der Schmerz, der in ihr schwelte, ohne je hervorzubrechen. Nichts ist verheilt. Nichts ist endgültig vorbei.“ In großer Monotonie verbringt sie ihr derzeitiges Leben: „Ist sie nicht mit so alltäglichen Dingen beschäftigt, sitzt sie bewegungslos auf dem Sofa“ oder sie „läuft bis zur Erschöpfung […] damit sie nicht durch einen Alptraum aufwacht, der sie dann davon abhalten würde weiterzuschlafen. Damit sie nicht bis zum Morgengrauen zwanghaft die Erinnerungsfetzen zusammensetzt, die in ihrem Kopf herumschwirren.“ In einem chronologisch noch weiteren Rückgriff um etwa zwanzig Jahre in das 17. Lebensjahr der Protagonistin wird von einer damals erstmalig aufgetretenen mutistischen Episode berichtet: „Die Wörter […] verschwanden plötzlich im Nichts. […] Die Erinnerung daran, wie die Zunge und die Lippen Laute formten […] war auf einmal nicht mehr greifbar. […] Kein Mitschüler interessierte sich für das Mädchen, das von früh bis spät schwieg. Wenn sie aufgerufen wurde, etwas vorzulesen, […] starrte sie nur stumm in das Gesicht des jeweiligen Lehrers. […] Sie kam nach Hause, fast schwebend, als bewege sie sich in einer riesigen Seifenblase durch eine Menschenmenge. […] Sie fühlte sich, als befände sie sich unter Wasser und betrachte von dort das Treiben an der Oberfläche.“ Weiterhin erfahren wir, dass sich das „sehr intelligente“ Kind bereits mit vier Jahren selbst die koreanische Schrift beigebracht hatte und sich stundenlang mit Konsonanten und Vokalen beschäftigte, Entdeckungen, „die sie in Aufregung und Verzückung“ versetzten. Sie habe immer schon leise gesprochen, und „obwohl sowieso schon zierlich, verringerte sie ihr Körpervolumen noch, indem sie die Schultern nach vorne zog und einen Buckel machte. Sie war witzig, zeigte auch oft ein freundliches Lächeln, doch wenn sie lachte, dann leise, kaum hörbar.“ Auch als sie noch sprechen konnte, „kam es vor, dass sie ihren Gesprächspartner einfach nur anstarrte. […] Sie grüßte, bedankte und entschuldigte sich mit ihren Augen und nicht mit Worten. Sie war der Ansicht, kein Kontakt könne so direkt und klar verständlich sein wie ein Blick. Für sie war das, abgesehen von einer Berührung, der einzige Weg zu kommunizieren. Verglichen damit machte ihr die Sprache wahnsinnige körperliche Mühe.“

So wie sich die Protagonistin selbst gegen die Deutungen ihres Psychotherapeuten verwehrt („Es ist nicht so einfach“) und nicht „als Fallbeispiel in einem Buch“ untergebracht werden möchte, klärt auch der Roman nicht restlos auf und nimmt keine diagnostische Einordnung vor. Dennoch liest man aus fachlicher Sicht die hier dargestellte Symptomkonstellation automatisch auch mit einem diagnostischen Blick und würde sie formal vielleicht zunächst als dissoziative Bewegungsstörung (ICD-10: F44.4) in Form einer psychogenen Aphonie interpretieren, zumal keine Hinweise auf ein organisches Korrelat der Störung vorliegen. Dabei wäre der relativ plötzliche sprachliche Funktionsverlust als Ausdruck emotionaler Konflikte oder Bedürfnisse bei psychischer Belastung nach Tod der Mutter und Verlust des Sorgerechts für den Sohn zu verstehen – hier verschlägt es der Protagonistin sozusagen vor Schreck, Angst und Trauer die Sprache, und die Lebenssituation fühlt sich „un-sag-bar“ schrecklich an. Auch eine bereits im 17. Lebensjahr erstmalig stattgehabte und zwischenzeitlich remittierte, mit einer Art Derealisationserleben verbundene mutistische Symptomatik könnte vor dem Hintergrund belastender familiärer Konstellationen gedeutet werden. Historisch würde man von einem psychogenen oder „hysterischen“ Mutismus im Sinne einer Konversionsstörung sprechen [8]. Wie vom französischen Psychiater Pierre Janet schon 1909 beschrieben [4], kann es hier zu einer vollständigen Sprachhemmung, aber auch logorrhoischen Krisen kommen – dazu passend redete die Hauptfigur im Buch intermittierend „paradoxerweise wie ein Wasserfall. Sie gab dann lange, grammatikalisch perfekte Schachtelsätze von sich“. Ein selektiver Mutismus, der nach DSM‑5 und ICD-11 taxonomisch zu den Angsterkrankungen gezählt wird [7], liegt strenggenommen nicht vor, da sich die mutistische Symptomatik nicht nur auf bestimmte Situationen beschränkt, sondern generalisiert besteht, wobei sich die Erkrankung gerade im Erwachsenenalter aber durchaus auch als totaler Mutismus äußern kann. Theoretische Differenzialdiagnosen des Mutismus, für die hier aber kein Anhalt besteht, wären zum Beispiel ein depressiver Stupor, eine katatone Schizophrenie, Taubheit, verschiedene neurologische bzw. Hals-Nasen-Ohren-Erkrankungen, die zu einer motorischen Aphasie oder Aphonie führen, der akinetische Mutismus, der durch eine schwerwiegende Störung des Antriebs und der Motorik im Allgemeinen gekennzeichnet ist, oder ein postoperativer zerebellärer Mutismus [1, 10]. Komorbid könnte bei der Protagonistin eine depressive Episode mit Antriebslosigkeit und psychomotorischer Unruhe im Wechsel, Grübelneigung, Schlafstörungen und Albträumen bestehen. Vor dem Hintergrund der Entwicklungsgeschichte mit der womöglich im Sinne eines Sonderinteresses oder einer Spezialbegabung zu interpretierenden, fast obsessiven Beschäftigung des vierjährigen Kindes mit Sprache könnte differenzialdiagnostisch auch ein zugrunde liegender hochfunktionaler Autismus erwogen werden, wobei die Betonung von Blickkontakt und Berührung in der Kommunikation eher dagegenspricht. Sozial-phobische Züge oder eine ängstlich-vermeidendende Persönlichkeitsakzentuierung könnten aus den Berichten aus der Kindheit der Protagonistin herausgelesen werden, die in der aktuellen Psychopathologie aber nicht im Vordergrund stehen.

Der psychogene Mutismus wird primär kognitiv-verhaltenstherapeutisch oder psychodynamisch-psychotherapeutisch behandelt [2, 9]. Die Ansprechraten sind hoch, die Erkrankung hat einen günstigen Verlauf, wobei Rezidive nicht selten sind [8]. Die Therapie eines Mutismus im Sinne einer Angststörung erfolgt ebenfalls vornehmlich psychotherapeutisch, teilweise auch gemeinsam mit weiteren Disziplinen wie z. B. Sprach- und Logotherapie. Das Konzept der kooperativen Mutismustherapie (KoMut) verfolgt einen systemisch-handlungsorientierten Ansatz und richtet sich vornehmlich an Kinder mit (selektivem) Mutismus [3]. Zudem kann der Kontakt zur Mutismus-Selbsthilfe (www.mutismus.de) sinnvoll sein.

Die Therapie der Hauptfigur in Han Kangs Roman besteht in der behutsamen emotionalen und körperlichen Annäherung an ihren langsam erblindenden Griechischlehrer, der ohne zu ahnen, dass „sie ihre bläulich-violetten Lippen fest geschlossen hält, weil all dies ihr Angst macht“, den Panzer der Stille aufzubrechen in der Lage ist: „Ich suchte nach Ihrer Schulter, um sie zu küssen. […] und Ihnen entschlüpfte ein winziger Laut. Zum ersten Mal, zart und rund wie eine Seifenblase“.
